# The Impact of ALS-Associated Genes *hnRNPA1*, *MATR3*, *VCP* and *UBQLN2* on the Severity of TDP-43 Aggregation

**DOI:** 10.3390/cells9081791

**Published:** 2020-07-28

**Authors:** Ana Bajc Česnik, Helena Motaln, Boris Rogelj

**Affiliations:** 1 Department of Biotechnology, Jozef Stefan Institute, 1000 Ljubljana, Slovenia; ana.bajc.cesnik@ijs.si (A.B.Č.); helena.motaln@ijs.si (H.M.); 2 Graduate School of Biomedicine, Faculty of Medicine, University of Ljubljana, 1000 Ljubljana, Slovenia; 3 Biomedical Research Institute BRIS, 1000 Ljubljana, Slovenia; 4 Faculty of Chemistry and Chemical Technology, University of Ljubljana, 1000 Ljubljana, Slovenia

**Keywords:** ALS, TDP-43, *hnRNPA1*, *MATR3*, *VCP*, *UBQLN2*, cytoplasmic aggregation

## Abstract

Amyotrophic lateral sclerosis is a progressive neurodegenerative disorder, characterized by cytoplasmic inclusions of RNA-binding protein TDP-43. Despite decades of research and identification of more than 50 genes associated with amyotrophic lateral sclerosis (ALS), the cause of TDP-43 translocation from the nucleus and its aggregation in the cytoplasm still remains unknown. Our study addressed the impact of selected ALS-associated genes on TDP-43 aggregation behavior in wild-type and aggregation prone TDP-43 in vitro cell models. These were developed by deleting TDP-43 nuclear localization signal and stepwise shortening its low-complexity region. The SH-SY5Y cells were co-transfected with the constructs of aggregation-prone TDP-43 and wild-type or mutant ALS-associated genes *hnRNPA1*, *MATR3*, *VCP* or *UBQLN2*. The investigated genes displayed a unique impact on TDP-43 aggregation, generating distinct types of cytoplasmic inclusions, similar to those already described as resembling prion strains, which could represent the basis for neurodegenerative disease heterogeneity.

## 1. Introduction

Amyotrophic lateral sclerosis (ALS) is a fatal progressive neurodegenerative disease pathologically characterized by cytoplasmic deposits of misfolded proteins in the affected neurons. The main component of these inclusions represents ubiquitinated, phosphorylated and cleaved TAR DNA-binding protein 43 (TDP-43) [1]. Under physiological conditions, TDP-43 is a predominantly nuclear protein, involved in various RNA processes, and comprises a *N*-terminal oligomerization domain, a classical bipartite nuclear localization signal (NLS), two RNA recognition motifs (RRM1 and RRM2) and a low-complexity domain (LCD) [2,3,4,5,6]. While 5–10% of ALS cases are considered to be familial, mutated TDP-43 is very rare, meaning that most of the patients with TDP-43 positive aggregates do not carry any mutation in this protein [7,8,9]. So far, more than 50 potentially causative or ALS-modifying genes have been identified that are mainly involved in two cellular processes: RNA metabolism (TDP-43, FUS, MATR3, hnRNPA1, hnRNPA2B1, TIA1, etc.) and quality control of protein metabolism (VCP, UBQLN2, SQSTM1, OPTN, etc.) [7,10,11,12,13,14,15,16,17,18,19,20,21]. The accumulation of misfolded proteins and their clearance both seem to have a similar deleterious effect on these two processes, therefore suggesting a common pathogenic cascade [22]. Still, an interplay of genetic and environmental factors rather than a single initiating event is thought to contribute to disease development and progression [23].

Recently, a liquid-liquid phase separation (LLPS) of RNA-binding proteins, such as TDP-43, has been implicated as a possible inception mechanism for protein aggregation and subsequent self-templating, ultimately resulting in various types of irreversible inclusions, i.e., amyloid-like fibrils in neurodegenerative disease [24]. The main drivers of LLPS in proteins are their LCDs and RRMs [25]. One of the proteins that readily undergoes LLPS via self-associating interactions through LCD is TDP-43 [26,27]. Its LCD can be divided into three segments: two intrinsically disordered regions (IDR1 and IDR2) interspaced with a conserved region (CR) prone to adapt α-helical fold [28]. Hydrophobic intermolecular interactions between α-helical regions of TDP-43 are thought to drive its LLPS [28,29,30]. The role of *N*-terminal domain in TDP-43 aggregation is somewhat controversial: while some argue that the oligomerization of TDP-43 via its *N*-terminal domain allows interactions with other partner proteins and RNA, thereby antagonizing its pathological aggregation, others suggest that it enhances the propensity of the intrinsically disordered *C*-terminal region towards aggregation [31,32]. It has been shown that the disruption of the *N*-terminal domain reduces propensity of TDP-43 to undergo LLPS and to form aggregates [33,34]. Moreover, TDP-43 contains two highly conserved RRMs, capable of binding to single strand RNA or DNA, which in turn enhances its solubility and prevents its aggregation [35,36,37,38,39].

Mounting evidence suggests that cytoplasmic accumulations of TDP-43 exhibit prion-like characteristics [40]. In addition to seeding and intracellular propagation of TDP-43 aggregations between cells in vitro and in vivo, different types of the TDP-43 aggregates in ALS and frontotemporal lobar degeneration (FTLD) diseased brains have been identified [41,42,43,44,45]. It has been proposed that alternate pathological conformations may form the basis for the diversity of TDP-43 proteinopathies and disease heterogeneity, reminiscent of prion strains [45]. There is some reported difference between aggregate deposits in FTLD, with mutations in progranulin and C9orf72 [46]. However, the role of ALS-associated genes, the possible impact of their mutations on TDP-43 aggregates behavior and properties remains largely unknown. Hence, in this study, we sought to compare the impact of several wild-type and mutated ALS-associated genes on TDP-43 aggregation in vitro.

First, we developed a novel in vitro TDP-43 aggregation model in the neuroblastoma SH-SY5Y cell line. To achieve TDP-43 cytoplasmic aggregation, we eliminated NLS from the full-length TDP-43 sequence. Then, we stepwise shortened its LCD (containing IDR and CR domains), to disrupt the physiological conformation of TDP-43 dimers. We left the *N*-terminal region of TDP-43 intact, as it is necessary for its oligomerization and phase-separation, although some studies have achieved TDP-43 aggregation with only the *C*-terminal region present [47,48]. Full-length TDP-43 lacking only NLS (dNLS) and TDP-43 without NLS and IDR2 (dNLSd343) constructs were used in co-transfection experiments, to assess the impact of wild-type and mutant ALS-associated genes on TDP-43 aggregate behavior. Heterogeneous nuclear ribonucleoprotein A1 (hnRNPA1), matrin-3 (MATR3), valosin-containing protein (VCP) and ubiquilin-2 (UBQLN2) were selected for their involvement in RNA metabolism, or as a part of protein turnover machinery. Our results confirm the unique influence of these genes and their ALS-related mutants on TDP-43 aggregation behavior, suggesting a possible origin for ALS protein inclusions heterogeneity, and different modes of disease progression.

## 2. Materials and Methods

### 2.1. Plasmids

TDP-43 constructs: TDP-43 was subcloned from previously published plasmid [49] into mKO2-C1 plasmid, containing sequence for fluorescent protein mKusabira Orange2 (mKO2) (Addgene #54494, deposited by Michael Davidson and Atsushi Miyawaki [50]). Primers (sequences are listed in Supplementary Data, Table S1) were ordered from IDT, United States, and Phusion™ High-Fidelity DNA Polymerase (Thermo Fisher Scientific, United States) was used for PCR reaction. Both TPD-43 insert and pKO2-C1 plasmid were digested with SalI and BamHI (NEB, United States) according to the manufacturer’s instructions and ligated at 16 °C overnight with T4 DNA Ligase (NEB). Plasmids were purified from bacterial culture with NucleoSpin Plasmid kit (Macherey-Nagel, Germany). Later, pcDNA5/FRT/TO backbone (Thermo Fisher Scientific) was selected. mKO2-TDP-43 sequence was inserted into pcDNA5/FRT/TO after its linearization with HindIII in NotI (Thermo Fisher Scientific) by CloneEZ PCR Cloning Kit (GenScript, United States) (primer sequences are listed in Supplementary Data, Table S2). Site-directed mutagenesis (KOD Hot Start polymerase, Sigma Aldrich, United States) was performed to eliminate NLS sequence from TDP-43 (82 a.a. to 98 a.a.), and to subsequently introduce stop codons to get TDP-43 fragments shortened at C-terminus (primer sequences are listed in Supplementary Data, Table S3). For control experiments, pcDNA5/FRT/TO plasmid containing only mKO2 sequence was used.

ALS-linked genes: First, a gBlock DNA fragment (IDT) containing HRV 3C site, Gly-Ser-Gly linker and 3xHA was amplified by Phusion™ High-Fidelity DNA Polymerase (primer sequences are listed in Supplementary Data, Table S4) and inserted into linearized pcDNA/FRT/TO plasmid (ApaI (NEB) and XhoI (NEB)), by CloneEZ PCR Cloning Kit. Then, four ALS-linked genes were amplified by Phusion™ High-Fidelity DNA Polymerase and inserted into linearized pcDNA5/FRT/TO-3xHA (XhoI (Thermo Fisher Scientific) and EcoRV (Thermo Fisher Scientific)) by CloneEZ PCR Cloning Kit: VCP (Addgene #23971, deposited by Nico Dantuma [51]), UBQLN2 (Addgene #8661, deposited by Peter Howley [52]), hnRNPA1 (Addgene #23026, deposited by Douglas Black, unpublished), MATR3 (Addgene #32880, deposited by Yossi Shiloh [53]) (primer sequences are listed in Supplementary Data, Table S5). To introduce ALS-linked mutations, site-directed mutagenesis with KOD Hot Start polymerase was performed (primer sequences are listed in Supplementary Data, Table S6). For control experiments, a start codon was introduced by site directed mutagenesis (KOD Hot Start polymerase) into pcDNA5/FRT/TO-3xHA plasmid (primer sequences are listed in Supplementary Data, Table S7), to get a 3xHA sequence with additional 96 nucleotide expressed after transfection. All plasmid constructs used in this study were confirmed by sequencing (Eurofins Genomics, Luxembourg).

### 2.2. Cell Culture

Neuroblastoma cells SH-SY5Y (ATCC^®^ CRL-2266™) were cultured as a monolayer in DMEM/F-12 (Thermo Fisher Scientific), supplemented with 10% (*v*/*v*) FBS (Thermo Fisher Scientific) and penicillin-streptomycin solution (Thermo Fisher Scientific) at 37 °C, in a humidified atmosphere containing 5% CO_2_. For immunocytochemistry, 1.1 × 10^5^ cells/well were plated on coverslips into 24-well plates, 24 h before transfection. For western blot analysis, 2.2 × 10^5^ cells/well were plated into 12-well plates and 6.5 × 10^5^ cells/well were plated on 6-well plates, 24 h before transfection. Cells were transiently transfected using Xfect transfection reagent according to manufacturer instructions (Takara Bio, Japan). Co-transfection experiments were carried out with plasmids DNA in ratios 1:1 amounting to 1 µg total DNA/well for 24-well plates, 2.5 µg total DNA/well for 12-well plates and 7.5 µg total DNA/well for 6-well plates. All of the co-transfections were carried out side-by-side at the same time, to allow comparisons between different experimental conditions.

### 2.3. Western Blot

For total cell lysates, 24 h after transfection, cells in 12-well plates were washed 3x with ice-cold PBS, and lysed in 2x SDS loading buffer with 0.1 M DTT. Samples were boiled at 100 °C for 10 min. For RIPA-soluble fraction, cells in 6-well plates were washed 3x with ice-cold PBS and lysed in 200 µL RIPA buffer (50 mM Tris pH 8.0, 150 mM NaCl, 1% NP-40, 5 mM EDTA, 0.5% sodium deoxycholate, 0.1% sodium dodecyl sulfate (SDS), containing protease inhibitors (cOmplete ULTRA tablets mini, EDTA-free EASYpack, Roche, Switzerland) and phosphatase inhibitors (PhosSTOP EASYpack, Roche, Switzerland). Samples were sonicated using an ultrasonic probe (cycle 0.5, amplitude 80%, three times 15 s interval—samples were cooled on ice during intervals) and centrifuged at 21,000× *g* for 30 min at 4 °C. The supernatants were saved for analysis as RIPA-soluble fractions and pellets were washed 3x in 200 µL RIPA buffer and centrifuged at 21,000× *g* for 30 min at 4 °C each time. After the last wash, 20 µL of UREA buffer (7 M urea, 2 M thiourea, 4% CHAPS, and 30 mM Tris, pH 8.5) was added to the pellets. The pellets were resuspended by pipetting and centrifuged again at 21,000× *g* for 30 min at 22 °C. The supernatants were collected as an UREA-soluble fraction. To both RIPA- and UREA-soluble fractions, the 6x SDS loading buffer with 0.3 M DTT was added, and RIPA-soluble fractions were boiled at 100 °C for 10 min. All samples were loaded to Novex WedgeWell 8–16% Tris-Glycine gels (Invitrogen Thermo Fisher Scientific) and run in SDS running buffer at 120 V for 90 min. Proteins were transferred onto Trans-Blot Turbo mini-size nitrocellulose membrane (Bio-Rad, United States) by Trans-Blot Turbo Transfer System (Bio-Rad) in Trans-Blot Turbo Transfer buffer (Bio-Rad). Membranes were washed once in TBS, blocked in 5% skim milk in 0.1% TBST for 30 min, and then incubated overnight with primary antibodies in 5% skim milk in 0.1% TBST (rabbit polyclonal anti-TDP-43 1:3000, #10782-2-AP Proteintech, United States; mouse anti-HA 1:5000, #HA-7 Sigma; mouse anti-GAPDH 1:5000, #60004-1-Ig Proteintech; rabbit polyclonal anti-GAPDH 1:5000, #10494-1-AP Proteintech). The next day, the membranes were washed 3 times in 0.1% TBST and incubated with secondary antibodies in 5% skim milk in 0.1% TBST for 1.5 h (anti-Rabbit Alexa 488 Cell Signaling Molecular Probes; anti-Mouse Alexa 647 Cell Signaling Molecular Probes; anti-Rabbit Alexa 647 Cell Signaling Molecular Probes; anti-Mouse Alexa 488 Cell Signaling Molecular Probes, all 1:5000). Upon three washes in 0.1% TBST, the fluorescence signal was detected using Chemidoc (Bio-Rad).

### 2.4. Immunocytochemistry

Twenty-four hours after transfection, cells were washed 3x with PBS, fixed in 4% PFA for 30 min and washed with PBS again. Then, they were permeabilized with 0.1% TX-100 in PBS for 10 min, blocked in 3% BSA in PBS for 30 min and incubated with primary antibodies (anti-HA-tag (C29F4) rabbit polyclonal Cell Signaling #3724, 1:500), overnight at 4 °C. The next day, the coverslips were washed, incubated with secondary antibodies (anti Rb_Alexa647, 1:1000; Cell Signaling) for 2 h, incubated in DAPI solution (0.1 μg/mL) (Thermo Fisher Scientific) for 15 min, and mounted on microscope slides with ProLong™ Gold Antifade Mountant (Thermo Fisher Scientific).

### 2.5. Imaging and Statistical Analysis

Slides were analyzed by confocal microscopy (Zeiss, Germany), using ZEN software. Three experiments were performed in duplicates for each experimental condition, and a minimum of 15 visual fields, each containing between 50–70 cells from each duplicate were taken. Overall, between 550 and 3400 transfected cells were analyzed per each experimental condition. The percentage of transfected cells harboring TDP-43 aggregates was quantified by manual counting. A further analysis of the number of aggregates per cell and their size were performed with ImageJ Shape Descriptors plugin (Threshold: min. 164, max 255; Size: 3-infinity; Circularity: 0.00–1.00). All data were recorded as averages ± *SEM*. The difference between averages was tested by ordinary one-way ANOVA followed by Tukey multiple comparisons test, using GraphPad Prism 6. The *p*-value < 0.05 was considered statistically significant. * *p* < 0.05, ** *p* < 0.01, *** *p* < 0.001, **** *p* < 0.0001.

## 3. Results

### 3.1. TDP-43 Aggregation Is Affected by Shortening of the C-Terminal Domain

**Figure 1:**
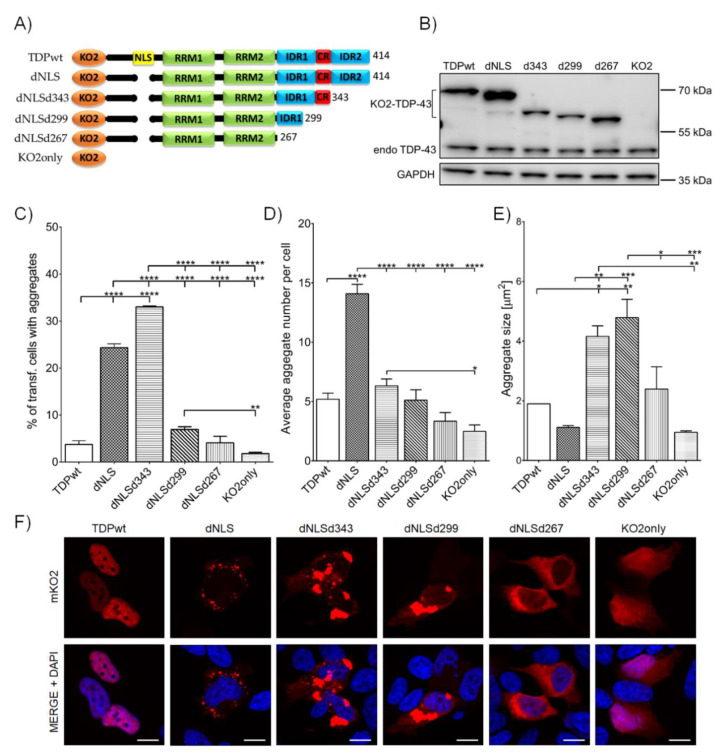
*C*-terminal domain truncations affect TDP-43 aggregate formation in the cytoplasm of SH-SY5Y cells: (**A**) A schematic representation of mKO2-tagged constructs and their abbreviations. Only TDPwt has nuclear localization signal (NLS), whereas others lack NLS and except dNLS that holds intact C-terminus, they carry deletions of LCD: IDR2 is deleted in dNLSd343 (ends at 343 aa residue), IDR2 and CR are deleted in dNLSd299 (ends at 299 aa residue); whole *C*-terminal domain is deleted in dNLSd267 (ends in 267 aa residues). A control construct KO2only holds sequence for mKO2 protein alone. (**B**) Western blot analysis of mKO2-tagged constructs. (**C**) Quantification of transfected cells harboring aggregates. (**D**) Average number of aggregates in an individual cell. (**E**) Average aggregate size within individual cells. (**F**) SH-SY5Y cells, expressing mKO2-tagged constructs. Nuclei are counterstained with DAPI. Scale bars: 10 µm.

To test the effect of *C*-terminal domain on TDP-43 aggregation, SH-SY5Y cells were transfected with various mKO2 tagged TDP-43 constructs, lacking NLS (dNLS) and different parts of LCD (dNLSd343, dNLSd299, and dNLSd267)—see Figure 1A. Wild-type TDP (TDPwt) was used to compare to the baseline aggregate formation of the wild-type TDP-43 protein and fluorescent protein mKO2 alone (KO2only); this was used as a control for the effect of the fusion protein on aggregation (Figure 1A). The expression of all constructs was detected with anti-TDP-43 antibody on western blot at predicted sizes (Figure 1B). As expected, the elimination of NLS in TDP-43 led to the increased cytoplasmic aggregation of dNLS (24.4 ± 0.80%) compared to wild-type TDP-43 protein (3.8 ± 0.80%) (Figure 1C). Moreover, a deletion of intrinsically disordered region 2 (IDR2) at the extreme C-terminus of TDP-43 (dNLSd343) resulted in an even higher percentage of transfected cells harboring dNLSd343 aggregates (33.0 ± 0.20%). On the contrary, the transfection of cells with constructs carrying *C*-terminal deletions of CR (dNLSd299) or IDR1 (dNLSd267) of TDP-43, decreased the percentage of cells with dNLSd299 (6.9 ± 0.57%) and dNLSd267 (4.1 ± 1.37%) aggregates. Further aggregate analyses within each individual cell revealed that the expression of dNLS resulted in the highest number of cytoplasmic aggregates per individual cell (14.1 ± 0.80), compared to the expression of any other TDP-43 truncated form (Figure 1D). However, the largest aggregates in size were noted in cells expressing dNLSd343 (4.2 ± 0.36 µm^2^) and dNLSd299 (4.8 ± 0.61 µm^2^) constructs (Figure 1E, F). Altogether, this implies that, besides the lack of NLS, which is enabling the cytoplasmic mislocalization of TDP-43, the elimination of specific regions of TDP-43 LCD may interfere with different stages of aggregate formation; either their initiation (aggregation proneness) or aggregation maturation.

### 3.2. The Mutations of ALS-Associated Genes Display Impact on TDP-43 Aggregation Behavior

The expression of both dNLS and dNLSd343 resulted in a highly increased percentage of cells exhibiting cytoplasmic aggregates, however, they clearly displayed a distinct number and size per individual cell (compare Figure 1B–D). Therefore, we decided to employ both of these TDP-43 aggregation models for further testing the effects of wild-type and mutated ALS-linked genes on their aggregation profile. For this reason, we co-transfected the SH-SY5Y cells with either TDPwt, dNLS, dNLSd343 or KO2only with selected wild-type (wt) or mutant (mut) ALS-linked gene tagged with 3xHA: *hnRNPA1*, *MATR3*, *VCP* or *UBQLN2*. These genes were selected based on their diverse roles in RNA processing and quality control of protein metabolism. A plasmid containing 3xHA with an additional 96 nucleotide sequence (pcDNA5/FRT/TO-3xHA - HAonly) was used as a baseline control of the aggregate formation (Supplementary Data, Figure S1).

#### 3.2.1. Overexpression of hnRNPA1 and Its D262V Mutation Inhibits TDP-43 Aggregate Formation and Their Maturation

**Figure 2:**
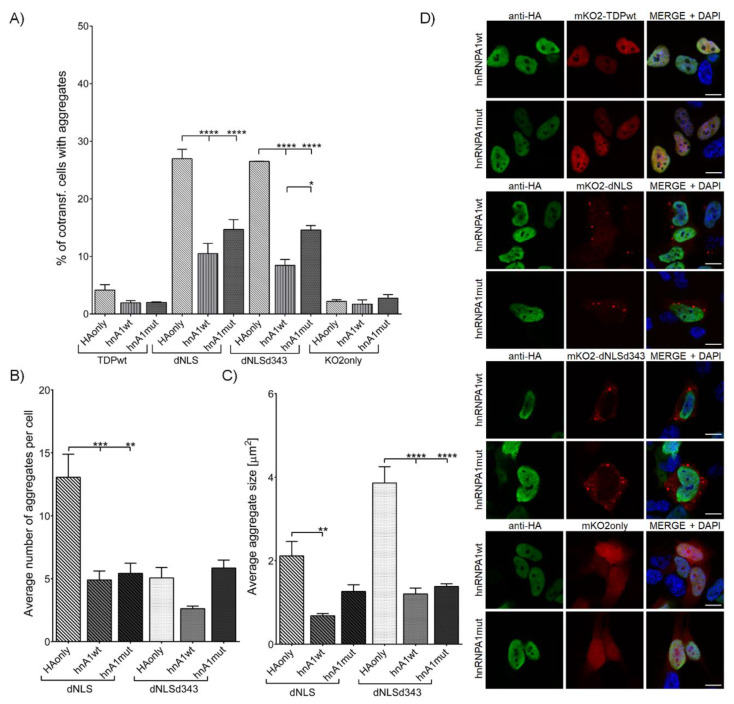
SH-SY5Y cells co-transfected with mKO2 constructs and wt or mut hnRNPA1. (**A**) Quantification of co-transfected cells harboring aggregates. (**B**) Average number of aggregates in an individual cell. (**C**) Average aggregate size. (**D**) SH-SY5Y cells co-transfected with mKO2 constructs and wt or mut hnRNPA1. Probed for HA-tag and counterstained with DAPI. Scale bars: 10 µm.

The co-transfection of dNLS and dNLSd343 constructs with either hnRNPA1wt or hnRNPA1mut (Supplementary Data, Figure S2A) resulted in a significantly lower percentage of transfected cells harboring aggregates. Though no difference in the percentage of cells harboring aggregates was noted between hnRNPA1wt (10.5 ± 1.77%) and hnRNPA1mut (14.7 ± 1.72%) co-transfected with dNLS, a co-transfection of hnRNPA1mut (14.6 ± 0.78%) with dNLSd343 led to an increased percentage of cells with aggregates as compared to hnRNPA1wt (8.5 ± 1.02%) co-transfection (Figure 2A). Additionally, the number of aggregates more than halved in the cells co-transfected either with hnRNPA1wt (4.9 ± 0.69) or hnRNPA1mut (5.4 ± 0.80) and dNLS, as compared to cells co-transfected with control plasmid HAonly (13.1 ± 1.82) (Figure 2B). Moreover, co-transfection of dNLS and dNLSd343 with hnRNPA1wt resulted in greatly decreased size of dNLS (0.7 ± 0.34 µm^2^) and dNLSd343 (1.2 ± 0.14 µm^2^) aggregates in comparison with the cells co-transfected with control HAonly (dNLS (2.1 ± 0.34 µm^2^) and dNLSd343 (3.9 ± 0.39 µm^2^)). The decreased aggregate size appeared more pronounced in case of dNLSd343 than dNLS cells co-transfected with either wild-type or mutant hnRNPA1 (Figure 2C,D). The resulting aggregates in all co-transfected cells were similar in size. This suggests, that IDR1 and CR domains of TDP-43 suffice for intermolecular interactions with hnRNPA1, which may play part in the inhibition of TDP-43 aggregate formation and maturation. Additionally, only the co-transfection of dNLS and hnRNPA1mut produced some insoluble aggregates, proved by dNLS detection in UREA-soluble fraction with Western blot (Supplementary Data, Figure S3A).

#### 3.2.2. MATR3 S85C Mutation Promotes TDP-43 Aggregation

**Figure 3:**
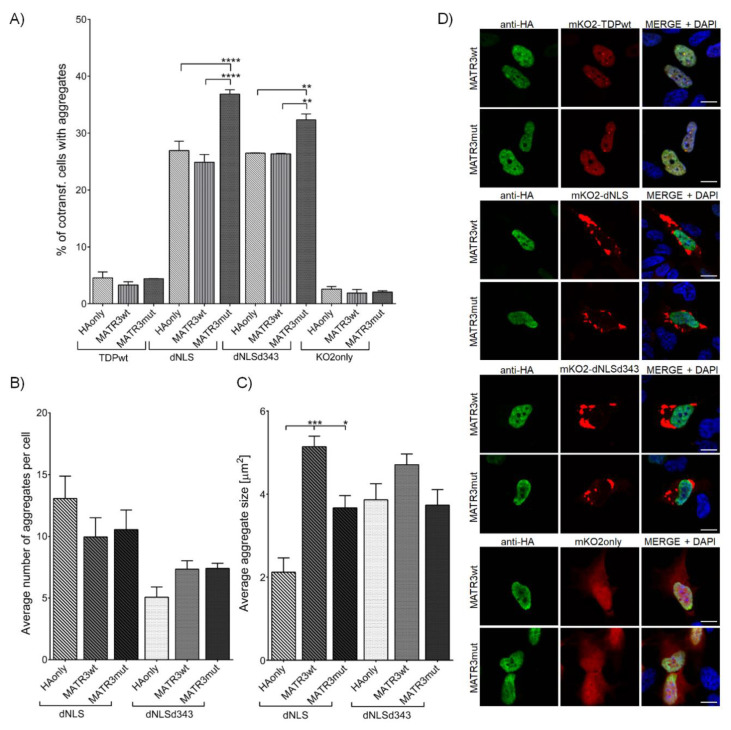
SH-SY5Y cells co-transfected with mKO2 constructs and wt or mut MATR3. (**A**) Quantification of co-transfected cells harboring aggregates. (**B**) Average number of aggregates in an individual cell. (**C**) Average aggregate size. (**D**) SH-SY5Y cells co-transfected with mKO2 constructs and wt or mut MATR3. Probed for HA-tag and counterstained with DAPI. Scale bars: 10 µm.

Co-transfection of either dNLS or dNLSd343 with MATR3wt (Supplementary Data, Figure S2B) had no effect on the percentage of cells with dNLS (24.9 ± 1.34%) or dNLSd343 (26.5 ± 0.10%) aggregates. On the other hand, MATR3mut co-transfected with either dNLS or dNLSd343 significantly increased the percentage of cells harboring both dNLS (36.9 ± 0.75%) and dNLSd343 (32.3 ± 1.02%) aggregates (Figure 3A). However, while neither MATR3wt nor MATR3mut displayed an effect on the number of aggregates within an individual cell (Figure 3B), both MATR3wt (5.1 ± 0.26 µm^2^) and MATR3mut (3.7 ± 0.30 µm^2^) evidently increased the size of aggregates in cells co-transfected with dNLS (Figure 3C,D). This was also confirmed by the detection of dNLS in UREA-soluble fraction with Western blot, demonstrating that the co-transfection of MATR3wt and dNLS produces more insoluble aggregates than the co-transfection of dNLS with MATR3mut in the cells (Supplementary Data, Figure S3B). These results are implying that even though MATR3wt does not seem to promote aggregate formation to the extent that its mutated form does, it nonetheless similarly promotes TDP-43 dNLS aggregate maturation, irrespective of TDP-43 *C*-terminal truncations.

#### 3.2.3. VCP R191Q Mutation Promotes TDP-43 dNLS but Not dNLSd343 Aggregate Maturation

**Figure 4:**
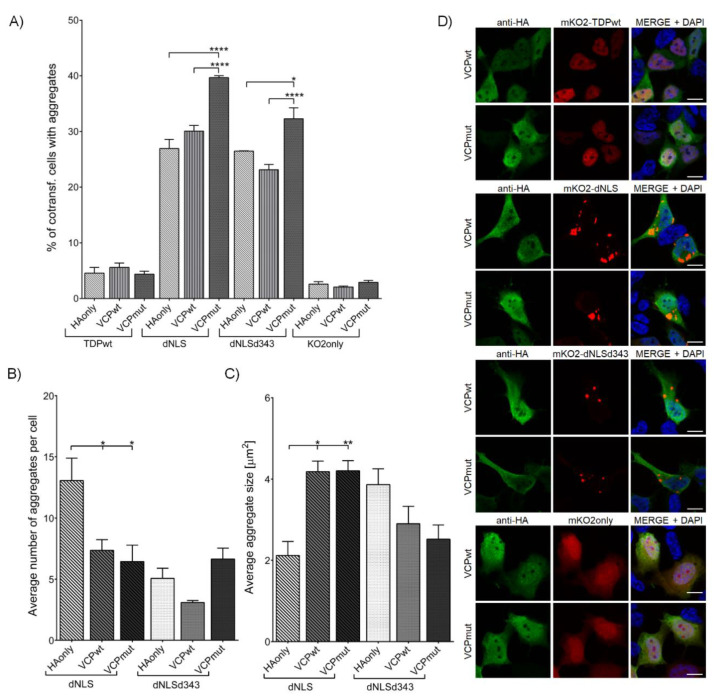
SH-SY5Y cells co-transfected with mKO2 constructs and wt or mut VCP. (**A**) Quantification of co-transfected cells harboring aggregates. (**B**) Average number of aggregates in an individual cell. (**C**) Average aggregate size. (**D**) SH-SY5Y cells co-transfected with mKO2 constructs and wt or mut VCP. Probed for HA-tag and counterstained with DAPI. Scale bars: 10 µm.

The co-transfection of dNLS or dNLSd343 with VCPmut (Supplementary Data, Figure S2C) resulted in significantly increased percentage of cells harboring dNLS (39.7 ± 0.36%) and dNLSd343 (32.3 ± 1.97%) aggregates in comparison with the cells co-transfected with VCPwt (dNLS (30.1 ± 1.04%) and dNLSd343 (23.1 ± 0.95%)) or control plasmid HAonly (dNLS (27.0 ± 1.64%) and dNLSd343 (26.5 ± 0.05%)) (Figure 4A). However, the average number of dNLS aggregates in individual cell decreased in both VCPwt (7.4 ± 0.87) and VCPmut (6.4 ± 1.35) co-transfected cells, compared to HAonly co-transfected cells (13.1 ± 1.82) (Figure 4B). Nevertheless, the size of dNLS appeared increased in both VCPwt (4.2 ± 0.25 µm^2^) and VCPmut (4.2 ± 0.24 µm^2^) co-transfected cells, compared to HAonly (2.1 ± 0.34 µm^2^) (Figure 4C,D). Additionally, the insolubility of dNLS aggregates was confirmed by Western blot detection of dNLS in UREA-soluble fraction (Supplementary Data, Figure S3C). No such distinct effect was noted in co-transfections of VCPwt (2.9 ± 0.43 µm^2^) and VCPmut (2.5 ± 0.35 µm^2^) with dNLSd343, suggesting the involvement of IDR2 domain of TDP-43 in VCP interactions, and its necessity for TDP-43 aggregate maturation.

#### 3.2.4. Wild-Type UBQLN2, but Not Its P506T Mutation Decreases Initiation of TDP-43 dNLS Aggregate Formation

**Figure 5:**
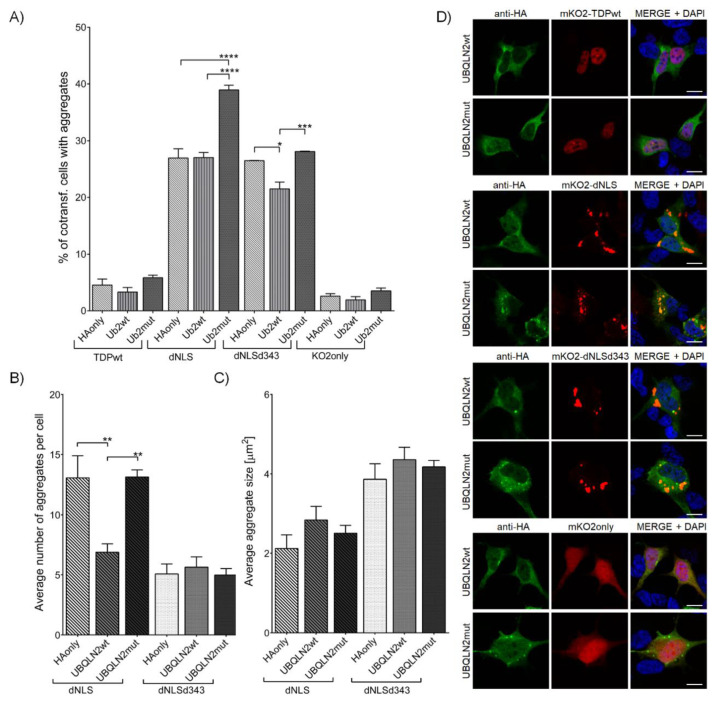
SH-SY5Y cells co-transfected with mKO2 constructs and wt or mut UBQLN2. (**A**) Quantification of co-transfected cells harboring aggregates. (**B**) Average number of aggregates in an individual cell. (**C**) Average aggregate size. (**D**) SH-SY5Y cells co-transfected with mKO2 constructs and wt or mut UBQLN2. Probed for HA-tag and counterstained with DAPI. Scale bars: 10 µm.

Similar to VCPmut, the co-transfection of TDP-43 dNLS construct with UBQLN2mut (Supplementary Data, Figure S2D) (39.0 ± 0.83%) led to an increased percentage of cells harboring aggregates in comparison to HAonly (27.0 ± 1.64%) or UBQLN2wt (27.0 ± 0.92%) (Figure 5A). On the contrary, the co-transfection of dNLSd343 with UBQLN2wt (21.5 ± 1.21%) resulted in the decreased percentage of cells containing aggregates, as compared to HAonly (26.5 ± 0.05%) or UBQLN2mut (28.1 ± 0.07%) co-transfected cells. UBQLN2 seems to have an impact on TDP-43 dNLS aggregate initiation, as the co-transfection of TDP-43 dNLS and UBQLN2wt (6.9 ± 0.70) extensively decreased the number of dNLS aggregates within the individual cells (Figure 5B), though with no effect on the dNLS aggregate size or intracellular distribution noted (Figure 5C,D). However, the higher percentage of cells with dNLS aggregates, combined with an increased number of aggregates in an individual cell co-transfected with UBQLN2mut and dNLS, resulted in a higher amount of RIPA-insoluble dNLS aggregates in comparison to co-transfection of dNLS and UBQLN2wt (Supplementary Data, Figure S3C).

## 4. Discussion

Despite the overwhelming majority of ALS cases being characterized by cytoplasmic deposits of TDP-43 protein in the affected cells, ALS patients display large phenotypic variability [46]. As was proposed for other neurodegenerative diseases, pathological and clinical heterogeneity could at least in part originate from distinct structural conformations of TDP-43 aggregates [54]. Indeed, TDP-43 deposits extracted from FTLD diseased brain displayed distinct densities, morphologies neurotoxicity and seeding, associated with disease duration and subtype [45]. However, studies discerning the impact of mutations of other ALS-associated genes on the behavior of TDP-43 aggregates, and consequently on the disease heterogeneity, are lacking.

Therefore, our study aimed to explore the influence of ALS-related genes and their mutations on aggregation pattern of TDP-43 in vitro. First, we developed a TDP-43 aggregation model in neuroblastoma SH-SY5Y cell line. To ensure cytoplasmic localization of TDP-43, we deleted NLS from the full-length sequence. As physiological TDP-43 oligomerization via its *N*-terminal domain spatially separates aggregation-prone LCD [31], we tried to disrupt the conformation of TDP-43 oligomers by truncating its *C*-terminal sequence. The TDP-43 fragments were designed based on the borders of TDP-43 LCD segments determined by Schmidt and colleagues [28]. Like others [34,55], we showed that full-length wild-type TDP-43 only rarely forms aggregates when expressed in cultured cells. Moreover, in accordance with the literature [34], our expressed dNLS readily aggregated in the cytoplasm, forming numerous small aggregates, in about a quarter of transfected cells. However, dNLSd343 expression, despite lower expression levels, caused inclusion formation in a third of transfected cells, though at a reduced number compared to dNLS, yet with much larger inclusion average size. This observation suggests, that the elimination of IDR2 from TDP-43 changes its (dNLSd343) propensity for LLPS and aggregate formation, possibly driving the aggregate maturation into larger insoluble inclusions. The removal of CR in dNLSd299 in our study did not completely abolish aggregate formation, as approximately 7% of transfected cells exhibited few large inclusions. On the other hand, a deletion of the whole LCD in TDP-43 (dNLSd267) resulted in levels of aggregate formation similar to control cells.

Expression of TDP-43 dNLS and dNLSd343 exhibited two dissimilar yet distinct aggregation patterns: dNLS displayed numerous dispersed and small aggregates, whereas dNLSd343 displayed fewer large bulk aggregates. Therefore, we further investigated both of them in the context of the influence ALS-associated genes may have on TDP-43 aggregation. We selected four different genes involved in ALS pathogenesis, each of them with a distinct role within the cell. Four chosen ALS-associated genes in their wild-type and mutant form were co-expressed with TDP-43wt, dNLS, dNLSd343 and KO2only in SH-SY5Y cells. The expression of all transfected constructs was confirmed by Western blotting. Even though the co-transfections of ALS-associated genes’ constructs with dNLSd343 displayed expression at really low levels, as compared to other TDP-43 constructs, they produced higher percentage of transfected cells with aggregates.

Notably, hnRNPA1 is a member of the hnRNP protein family, involved in splicing, mRNA stabilization, miRNA biogenesis and transcriptional regulation [56,57,58,59]. Remarkably, the co-transfection of either hnRNPA1wt or mut with dNLS or dNLSd343 resulted in a decreased number of co-transfected cells, harboring either dNLS or dNLSd343 aggregates. The co-expression of both dNLS and dNLSd343 with hnRNPA1 in cells resulted in much less numerous, smaller and more rounded aggregates formation, suggesting that these proteins in a dense phase could be dynamically exchanged with the surrounding proteins in a more diluted phase. This observation was confirmed by the analyses of UREA-soluble fractions, where only dNLS in the cells co-transfected with dNLS and hnRNPA1mut was insoluble enough in RIPA buffer to be detected by Western blot. It has been shown that hnRNPA1 can form reversible amyloid fibrils described as dynamic states of protein assemblies with high free energies [60]. Together with the fact that the *C*-terminal part of TDP-43 directly interacts with *C*-terminal domain of hnRNPA1 [61] and that they co-phase separate [62], we can speculate that their interactions promote more homogeneous dissolution of proteins with less dense dynamic structures. Moreover, a recent study of TDP-43 aggregates using atomic force microscopy has shown that TDP-43 fragments lacking *C*-terminal domain form smaller aggregates compared to a full-length protein [38]. This is consistent with the notion that TDP-43-hnRNPA1 intermolecular interactions via their *C*-terminal domains spatially separate aggregation-prone LCDs and reduce the number of large aggregates, as observed in our study. Interestingly, even though hnRNPA1 undergoes LLPS and can form irreversible fibrils, which are enhanced by ALS-linked mutation D262V [16,27,63], we could not detect any cytoplasmic accumulation of either hnRNPA1wt or mut by microscopy. There was, however, a difference in the number of cells containing aggregates between the cells co-transfected with TDP-43 dNLSd343 and hnRNPA1wt or hnRNPA1mut, indicating the promoting effect of hnRNPA1 D262V mutation on driving dNLSd343 LLPS towards aggregate formation.

MATR3 is also an RNA-binding protein, localized in the nuclear matrix and involved in various tasks concerning nucleic acids, such as chromatin organization, DNA replication, transcription and repair, RNA processing, transport, stability and alternative splicing [53,64,65,66,67]. Co-transfection of MATR3 bearing S85C mutation with either TDP-43 dNLS or dNLSd343 increased the number of cells with aggregates in comparison with MATR3wt or control plasmid co-transfections. As shown previously, lower levels of RNA promote LLPS of RNA-binding proteins [68] and TDP-43 assemblies lacking RNA are insoluble [37,62]. Expression of MATR3 with S85C mutation increases retention of mRNA in the nucleus [69], possibly leading to a higher tendency of TDP-43 dNLS and dNLSd343 to phase-separate, and tipping the balance towards their insoluble inclusions. Additionally, *N*-terminal sequence of MATR3 exhibits low complexity and is able to undergo phase separation to form liquid-like droplets in the nucleus [70,71]. The introduction of MATR3 S85C mutation inhibits droplet formation [71,72], possibly providing us with an explanation for the increased size of TDP-43 dNLS aggregates, especially in cells co-transfected with MATR3wt. Indeed, increased levels of dNLS in cells co-transfected with dNLS and MATR3wt detected in UREA-soluble fraction compared to cells co-transfected with dNLS and MATR3mut, corroborate this proposition.

In contrast to previously discussed proteins mainly associated with RNA processes, one of VCP’s main duties is facilitating the proteasomal degradation of damaged or misfolded proteins by binding to polyubiquinated proteins and delivering them to the proteasome complex [73,74,75]. Soluble TDP-43 is degraded primarily by the ubiquitin proteasome system, whereas the removal of TDP-43 aggregates requires autophagy [76]. Therefore, it is not surprising that co-expression of mutant VCP gene with TDP-43 dNLS and dNLSd343 resulted in an increased proportion of transfected cells with dNLS and dNLSd343 aggregates, respectively. The increased number of aggregates was possibly initiated by the interference of defective VCP in with the degradation pathway of mutated TDP-43. However, co-expressing VCPwt with TDP-43 dNLS and dNLSd343 did not reduce the percentage of transfected cells with aggregates, even though VCP is supposed to prevent aggregation [77], suggesting an upper limit of proteolytic machinery to degrade misfolded proteins. In addition to VCP role in protein clearance, other functions have also been ascribed to it, such as its involvement in cellular stress response, i.e., affecting stress granule assembly [78] and clearance [79,80]. Stress granules also assemble by LLPS [81] and are RNA-protein aggregates known to contain TDP-43 and other ALS-related proteins [82,83,84,85,86], that are thought to represent precursors of pathological cytoplasmic inclusions [87], similar as modelled by our constructs. Therefore, the role of VCP in stress granule disassembly could explain its inhibitory effect on the number and promoting effect on the size and insolubility of dNLS aggregates, in comparison with controls. Recently published data revealed that under hyperosmotic stress, VCP, along with other proteasome-interacting proteins forms nuclear proteasomal foci behaving as liquid droplets [88]. In light of this study, VCP could actively participate in altering the conditions for dNLS LLPS, and thus changing the dNLS aggregate properties.

Like VCP, UBQLN2 participates in proteolytic processes, such as delivering ubiquitinated proteins to the proteasome. Besides, it is also involved in autophagy, cell signaling, cell cycle progression and cytoskeletal association [89]. Similarly, co-transfection of cells with UBQLN2 carrying P506T mutation with TDP-43 dNLS resulted in increased percentage of aggregates in transfected cells, when compared to control or UBQLN2wt co-transfected cells. Probably, similar tampering of UBQLN2 mutant with TDP-43 degradation machinery took place. However, co-expressing TDP-43 dNLSd343 with UBQLN2wt led to a decreased percentage of transfected cells harboring aggregates, suggesting the possible ability of UBQLN2wt to boost the degradation of dNLSd343. In addition to its other functions, UBQLN2 associates with stress granule components and regulates their formation by delaying initiation processes [90]. Latter could explain the reduced number of aggregates in UBQLN2wt and TDP-43 dNLS co-transfected cells. Additionally, UBQLN2 itself undergoes LLPS [91] and its ALS-associated mutations change its LLPS properties, affect its solubility and promote its oligomerization [92]. This is consistent with the observed increased percentage of cells harboring aggregates, and their increased insolubility upon co-transfection of cells with UBQLN2mut and dNLS. The detected co-localization of dNLS aggregates with UBQLN2mut indicates possible reciprocal influence on their tendencies to phase separate. However, since UBQLN2mut had no effect on dNLSd343 aggregation pattern, this suggests that the lacking amino acid residues could be crucial for the cooperation of TDP-43 and UBQLN2 during LLPS and the initial stages of aggregate formation.

**Figure 6:**
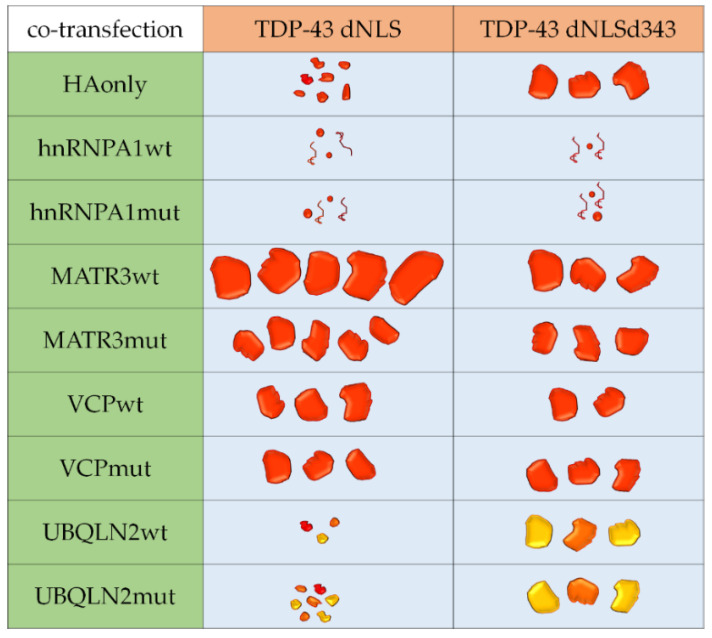
Summary of TDP-43 aggregation strains in co-transfected SH-SY5Y cells. Full red shapes represent TDP-43 aggregates, twisted lines represent soluble TDP-43, and orange and yellow shapes represent TDP-43 aggregates co-localized with UBQLN2.

Over the recent years, the extent of ALS heterogeneity, as well as its clinical, pathological and genetic overlap with several other degenerative disorders has become increasingly evident [93]. Nevertheless, research studies usually focus on individual genes or processes, neglecting the need to connect pathological pathways with the disease onset and progression. Our study shows that each of the investigated ALS-associated genes has a unique impact on TDP-43 aggregation, implicating the importance of their pathways on disease severity and progression (Figure 6). It could even be argued that each ALS-linked gene that we co-transfected with aggregation-prone construct led to the formation of distinct “aggregate type,” similar to those identified by Laferrière and colleagues [45], which could represent the basis for disease heterogeneity.

However, determining the influence of ALS-associated genes on the aggregation pattern of TDP-43 remains the first step in elucidating the mechanisms of TDP-43 aggregation. It would be important to confirm these differences in TDP-43 aggregation patterns directly in the aggregates extracted from post mortem tissue or from motor neurons derived from ALS patients, with distinct mutations in ALS-associated genes. Additional studies, revealing the effects of TDP-43 interactors with the potential to reduce or accelerate TDP-43 aggregation in combination with different mutations of ALS-associated genes, are definitely needed.

In summary, our results underline the significance of comparative research in the ALS field and provide an understanding of how different mechanisms interact at the molecular level and lead to neurodegeneration that may prove crucial in future for the successful development of therapeutics.

## Supplementary Materials

The following are available online at https://www.mdpi.com/2073-4409/9/8/1791/s1, Figure S1: TDP-43 constructs co-transfected with plasmid HAonly, Figure S2: Western blots of total lysates of SH-SY5Y cells co-transfected with KO2-TDP-43 constructs and HA-tagged wild-type and mutant ALS-associated genes, Figure S3: Solubility of TDPdNLS and TDPdNLSd343 constructs co-transfected in SH-SY5Y cells with HA-tagged wild-type and mutant ALS-associated genes, Figure S4: Original, uncropped and unadjusted images used for Figure 1, Figure S5: Original, uncropped and unadjusted images used for Figure S2A and S2B, Figure S6: Original, uncropped and unadjusted images used for Figure S2A and S2B, Figure S7: Original, uncropped and unadjusted images used for Figure S3, Table S1: Primer sequences for PCR to clone TDP-43 sequence into mKO2-C1 plasmid. Table S2: Primer sequences for PCR to clone mKO2-TDP-43 sequence into pcDNA5/FRT/TO plasmid. Table S3: Primer sequences for site-directed mutagenesis to produce dNLS *C*-terminally shortened TDP-43 fragments. Table S4: Primer sequences to amplify gBlock DNA fragment containing HRV 3C site, Gly-Ser-Gly linker, 3x HA tag. Table S5: Primer sequences for subcloning ALS-associated genes into pcDNA5/FRT/TO-3xHA. Table S6: Primer sequences for site-directed mutagenesis to introduce ALS-associated mutations. Table S7: Primer sequences used for site-directed mutagenesis to add start codon to pcDNA5/FRT/TO-3xHA.
