# Supplementary material for: The Impact of ALS-Associated Genes hnRNPA1, MATR3, VCP and UBQLN2 on the Severity of TDP-43 Aggregation
Source: Cells. 2020 Jul 28;9(8):1791. doi: 10.3390/cells9081791 (PMC7465640; doi:10.3390/cells9081791)
Supplement: Supplementary file 1 [file cells-09-01791-s001.pdf]

Supplementary data

Table S1: Primer sequences for PCR to clone TDP-43 sequence into mKO2-C1 plasmid.

Primer Name	Sequence
SalI koz TDP-43 Fw	AAA AGT CGA CGC CAC CAT GGC CTC TGA ATA TAT TCG GGT AAC
TDP-43 BamHI Rev	AAA GGA TCC GGC ATT CCC CAG CCA GAA GAC TTA G

Table S2: Primer sequences for PCR to clone mKO2-TDP-43 sequence into pcDNA5/FRT/TO plasmid

Primer Name	Sequence
FRT KO2 Fw	GTT TAA ACT TAA GCT GCC ACC ATG GTG AGT GTG AT
TDP HA Rev	TAG ACT CGA GCG GCC TCC CTA AGC GTA ATC TGG AAC ATC GTA TGG GTA CAT TCC CCA GCC AGA AGA CTT AGA

Table S3: Primer sequences for site-directed mutagenesis to produce dNLS C-terminally shortened TDP-43 fragments. Nucleotide change sites are in small caps and underlined.

Primer Name	Sequence
TDP dNLS Fw	GTG TAT GTT GTC AAC TAT CCA AAA GAT AAC GCA GTC CAG AAA ACA TCC G
TDP dNLS Rev	CGG ATG TTT TCT GGA CTG CGT TAT CTT TTG GAT AGT TGA CAA CAT ACA C
TDPdNLS stop Fw	CTT CTG GCT GG GGA ATG tag TAC CCA TAC GAT GTT CCA G
TDPdNLS stop Rev	CTG GAA CAT CGT ATG GGT Act aCA TTC CCC AGC CAG AAG
TDP dNLSd343 Fw	ATG GGC ATG TTA GCC AGC CAG tAG AAC CAG TCA GGC CCA T
TDP dNLSd343 Rev	ATG GGC CTG ACT GGT TCT aCT GGC TGG CTA ACA TGC CCA T
TDP dNLSd299 Fw	CGA ACC TAA GCA CAA TAG CAA TtG ACA GTT AGA AAG AAG TGG AAG ATT TGG
TDP dNLSd299 Rev	CCA AAT CTT CCA CTT CTT TCT AAC TGT CaA TTG CTA TTG TGC TTA GGT TCG
TDP dNLSd267 Fw	GCA GAG GGG GTG GAG CTG GTT TGt GAA ACA ATC AAG GTA GTA ATA TGG GTG G
TDP dNLSd267 Rev	CCA CCC ATA TTA CTA CCT TGA TTG TTT CaC AAA CCA GCT CCA CCC CCT CTG C

Table S4: Primer sequences to amplify gBlock DNA fragment containing HRV 3C site, Gly-Ser-Gly linker, 3x HA tag and to clone into pcDNA5/FRT/TO plasmid

Primer Name	Sequence
HRV 3C site Fw	AGT GGC GGC CGC TCG AGG GAA GCG GCC TGG AAG TTC TGT
Rev HRV 3xHA	TCA GCG GGT TTA AAC GGG CCC CTA AGC GTA ATC TGG AAC GTC GTA

Table S5: Primer sequences for subcloning ALS-associated genes into pcDNA5/FRT/TO3xHA

Primer Name	Sequence
Fw MATR3 3xHA	GAA TTC TGC AGA TAT CAG CCA CCA TGG CGT CCA AGT CAT TCC AGC AG
Rev MATR3 3xHA	TCC AGG CCG CTT CCC TCG AGA CAT CCG CCA GTT TCC TTC TTC TGT CTG
FwhnRNPA1 3xHA	GAA TTC TGC AGA TAT CAG CCA CCA TGG CGT CTA AGT CAG AGT CTC CTA A
RevhnRNPA1 3xHA	TCC AGG CCG CTT CCC TCG AGA CAT CCG CCA AAT CTT CTG CCA CTG CCA
Fw VCP 3xHA	TGG AAT TCT GCA GAT ATC AGC CAC CAT GGC TTC TGG AGC CGA TT
Rev VCP 3xHA	TCC AGG CCG CTT CCC TCG AGA CAT CCG CCG CCA TAC AGG TCA TCA TC
Fw UBQLN2 3xHA	ATT CTG CAG ATA TCA GCC ACC ATG GCT GAG AAT GGC GAG A
Rev UBQLN2 3xHA	TCC AGG CCG CTT CCC TCG AGA CAT CCG CCC GAT GGC TGG GAG CCC AG

Table S6: Primer sequences for site-directed mutagenesis to introduce ALS-associated mutations. Nucleotide change sites are in small caps and underlined.

Primer Name	Sequence	AA change
Fw Matr3 C-G	TTC TTC CCA TAA TTT GCA GTg TAT ATT TAA CAT TGG AAG T	S85C
Rev Matr3 C-G	ACT TCC AAT GTT AA ATA Tac ACT GCA AAT TAT GGG AAG AA	
Fw hnRNPA1 A-T	GAG GTG GTG GAA GCT ACA ATG tTT TTG GGA ATT ACA ACA ATC	D262V
Rev hnRNPA1 A-T	GAT TGT TGT AAT TCC CAA AAa CAT TGT AGC TTC CAC CAC CTC	

Fw VCP G-A	CGA AGG GGA GCC TAT CAA Aca AGA GGA TGA GGA AGA GTC CT	R191Q
Rev VCP G-A	AGG ACT CTT CCT CAT CCT CTt GTT TGA TAG GCT CCC CTT CG	
Fw UBQLN2 C-A	CAT AGG CCC TAT AGT CaC TTT TAC CCC CAT A	P506T
Rev UBQLN2 C-A	TAT GG GGG TAA AAG tGA CTA TAG GGC CTA TG	

Table S7: Primer sequences used for site-directed mutagenesis to add start codon to pcDNA5/FRT/TO3xHA

Primer Name	Sequence
Fw3xHA Start	GCT CGG ATC CAC TAG GCC ACC ATG TCC AGT GTG GTG G
Rev 3xHA start	CCA CCA CAC TGG ACA TGG TGG CCT AGT GGA TCC GAG C

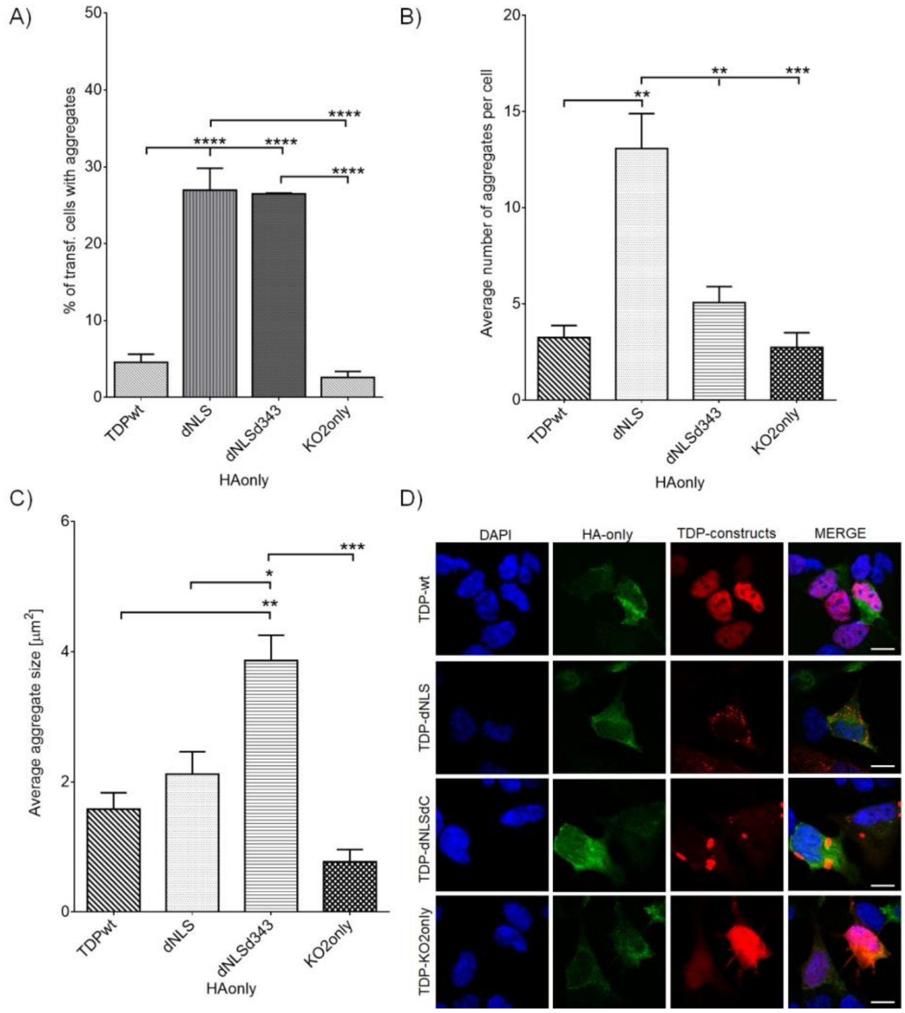

Figure S1: TDP-43 constructs co-transfected with plasmid HAonly. (**A**) Quantification of co-transfected cells harboring aggregates. TDPwt (4.6±1.05%), dNLS (27.0±1.64%), dNLSd343 (26.5±0.05%), and KO2only (2.6±0.45%). (**B**) Average number of aggregates in individual cells. TDPwt (3.2±0.63), dNLS (13.1±1.82), dNLSd343 (5.1±0.83), and KO2only (2.7±0.77). (**C**) Average aggregate size. TDPwt (1.6±0.25μm2), dNLS (2.1±0.34μm2), dNLSd343 (3.9±0.39μm2), and KO2only (0.77±0.19μm2). (**D**) SH-SY5Y cells co-transfected with mKO2-TDP-43 constructs and HAonly plasmid. Probed for HA-tag and counterstained with DAPI. Scale bars: 10 μm. All experiments were performed in duplicates and repeated three times. Data are presented as mean±s.e.m. *P<0.05, **P<0.01, ***P<0.001, ****P<0.0001; One-way ANOVA, Tukey post-hoc test.

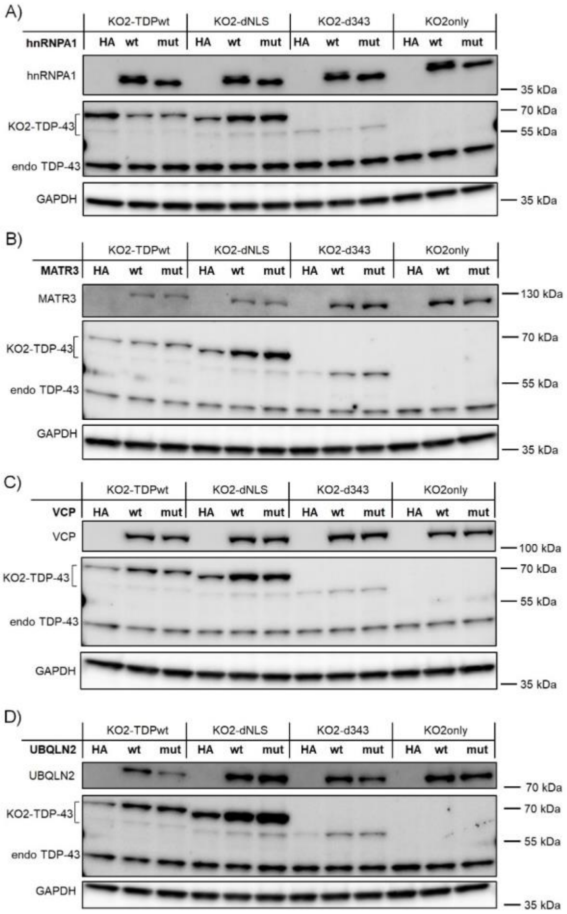

Figure S2: Western blots of total lysates of SH-SY5Y cells co-transfected with KO2-TDP-43 constructs and HA-tagged wild-type and mutant ALS-associated genes. (**A)** Co-transfection of wt or mut HA-tagged hnRNPA1 and TDPwt, dNLS, dNLSd343, or KO2only. (**B**) Co-transfection of wt or mut HA-tagged MATR3 and TDPwt, dNLS, dNLSd343, or KO2only. (**C**) Co-transfection of wt or mut HA-tagged VCP and TDPwt, dNLS, dNLSd343, or KO2only. (**D**) Co-transfection of wt or mut HA-tagged UBQLN2 and TDPwt, dNLS, dNLSd343, or KO2only. Plasmid containing only 3xHAtag was co-transfected with KO2-TDP-43 constructs as a baseline control. GAPDH was detected as a loading control.

Formatted: Not Highlight

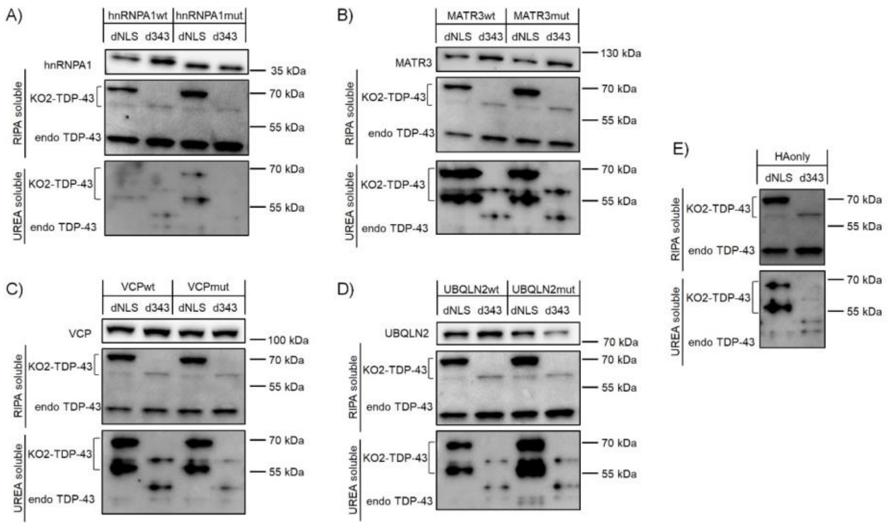

Figure S3: Solubility of TDPdNLS and TDPdNLSd343 constructs co-transfected in SH-SY5Y cells with HA-tagged wild-type and mutant ALS-associated genes. (**A)** RIPA soluble and UREA soluble fractions of SH-SY5Y cells co-transfected with wt or mut HA-tagged hnRNPA1 and TDPdNLS or dNLSd343. (**B**) RIPA soluble and UREA soluble fractions of SH-SY5Y cells co-transfected with wt or mut HA-tagged MATR3 and TDPdNLS or dNLSd343. (**C**) RIPA soluble and UREA soluble fractions of SH-SY5Y cells co-transfected with wt or mut HA-tagged VCP and TDPdNLS or dNLSd343. (**D**) RIPA soluble and UREA soluble fractions of SH-SY5Y cells co-transfected with wt or mut HA-tagged UBQLN2 and TDPdNLS or dNLSd343. (**D**) RIPA soluble and UREA soluble fractions of SH-SY5Y cells co-transfected with plasmid containing only 3xHAtag and TDPdNLS or dNLSd343.

Formatted: Not Highlight

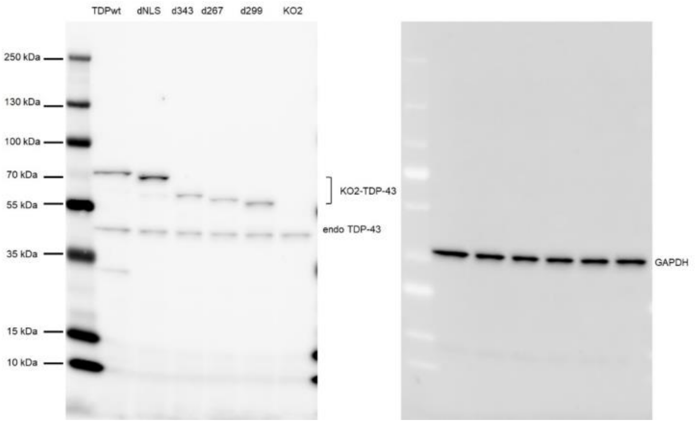

Figure S4: Original, uncropped and unadjusted images used for Figure 1. Membrane in first column: Primary antibody: Rabbit polyclonal anti-TDP-43 1:3000, #10782-2-AP Proteintech; Secondary antibody: anti-Rabbit Alexa 647 1:5000, Cell Signaling Molecular Probes. Membrane in second column: Primary antibody: mouse anti-GAPDH 1:5000, #60004-1-Ig Proteintech; Secondary antibody: anti-Mouse Alexa 488 1:5000, Cell Signaling Molecular Probes.

Formatted: Not Highlight

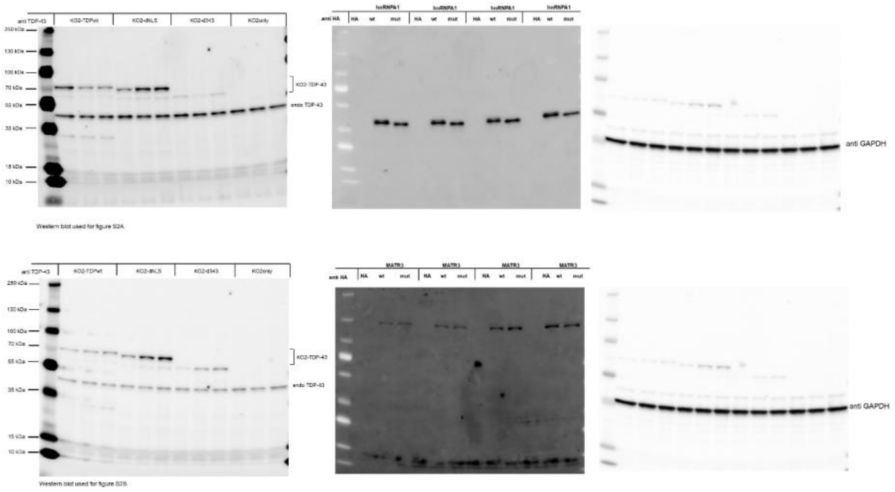

Figure S5: Original, uncropped and unadjusted images used for Figure S2A and S2B. Membranes in first column: Primary antibody: Rabbit polyclonal anti-TDP-43 1:3000, #10782-2-AP Proteintech; Secondary antibody: anti-Rabbit Alexa 647 1:5000, Cell Signaling Molecular Probes. Membranes in second column: Primary antibody: Mouse anti-HA 1:5000, #HA-7 Sigma; Secondary antibody: anti-Mouse Alexa 488 1:5000, Cell Signaling Molecular Probes. Membranes in third column: Membranes from the first column incubated with antibodies one more time. Primary antibody: Rabbit polyclonal anti-GAPDH 1:5000, #10494-1-AP Proteintech; Secondary antibody: anti-Rabbit Alexa 647 Cell Signaling Molecular Probes.

Formatted: Not Highlight

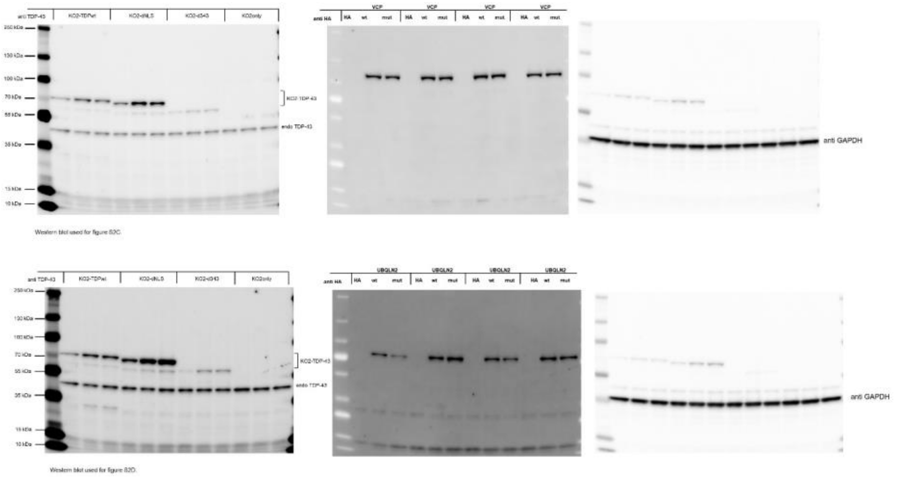

Figure S6: Original, uncropped and unadjusted images used for Figure S2A and S2B. Membranes in first column: Primary antibody: Rabbit polyclonal anti-TDP-43 1:3000, #10782-2-AP Proteintech; Secondary antibody: anti-Rabbit Alexa 647 1:5000, Cell Signaling Molecular Probes. Membranes in second column: Primary antibody: Mouse anti-HA 1:5000, #HA-7 Sigma; Secondary antibody: anti-Mouse Alexa 488 1:5000, Cell Signaling Molecular Probes. Membranes in third column: Membranes from the first column incubated with antibodies one more time. Primary antibody: Rabbit polyclonal anti-GAPDH 1:5000, #10494-1-AP Proteintech; Secondary antibody: anti-Rabbit Alexa 647 Cell Signaling Molecular Probes.

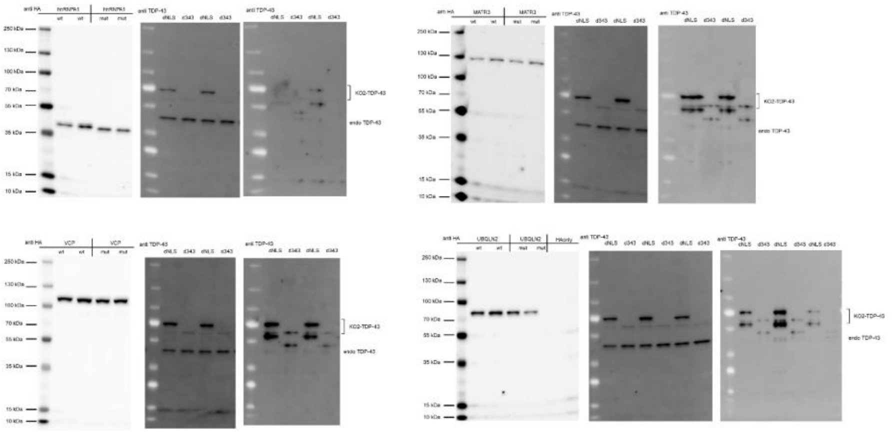

Figure S7: Original, uncropped and unadjusted images used for Figure S3. Membranes in first column: Primary antibody: Mouse anti-HA 1:5000, #HA-7 Sigma; Secondary antibody: anti-Mouse Alexa 647 1:5000 Cell Signaling Molecular Probes. Membranes in second and third column: Primary antibody: Rabbit polyclonal anti-TDP-43 1:3000, #10782-2-AP Proteintech; Secondary antibody: Anti-Rabbit Alexa 488 1:5000, Cell Signaling Molecular Probes.
